# A Human Gesture Mapping Method to Control a Multi‐Functional Hand for Robot‐Assisted Laparoscopic Surgery: The MUSHA Case

**DOI:** 10.3389/frobt.2021.741807

**Published:** 2021-12-10

**Authors:** Fanny Ficuciello, Alberto Villani, Tommaso Lisini Baldi, Domenico Prattichizzo

**Affiliations:** ^1^ Department of Electrical Engineering and Information Technology, University of Naples Federico II, Napoli, Italy; ^2^ Department of Information Engineering and Mathematics, University of Siena, Siena, Italy; ^3^ Department of Advanced Robotics, Istituto Italiano di Tecnologia, Genova, Italy

**Keywords:** medical robot, surgical gripper, robotics, surgical robot, teleoperation

## Abstract

This work presents a novel technique to control multi-functional hand for robot-assisted laparoscopic surgery. We tested the technique using the MUSHA multi-functional hand, a robot-aided minimally invasive surgery tool with more degrees of freedom than the standard commercial end-effector of the da Vinci robot. Extra degrees of freedom require the development of a proper control strategy to guarantee high performance and avoid an increasing complexity of control consoles. However, developing reliable control algorithms while reducing the control side’s mechanical complexity is still an open challenge. In the proposed solution, we present a control strategy that projects the human hand motions into the robot actuation space. The human hand motions are tracked by a LeapMotion camera and mapped into the actuation space of the virtualized end-effector. The effectiveness of the proposed method was evaluated in a twofold manner. Firstly, we verified the Lyapunov stability of the algorithm, then an user study with 10 subjects assessed the intuitiveness and usability of the system.

## 1 Introduction

In minimally invasive surgery (MIS), surgical instruments are introduced into the patient’s body through small incisions, this practice allows to reduce trauma. As scientific research has amply demonstrated, MIS offers several benefits such as reduced pain, limited post-operative course, and short patient recovery times (Perigli et al., 2008; Sood et al., 2017; Hanley et al., 2019; Chadi et al., 2020). On the other hand, 1) the surgeon loses dexterity due to the limited range of movement of the instruments and their limited number of degrees of freedom (DoFs), 2) there is a reduction of tactile sensations with a consequent risk of tissue damage, and 3) the hand-eye coordination decrease. Nowadays, according to Tonutti et al. (2017), such of these issues are addressed by the introduction of technology in surgical rooms. Indeed, robotics has proven to be a viable solution to mitigate the limitations associated with the MIS. Rapid advances in image systems and computer technologies have led to significant advances in robotics applied to medicine. Robot-assisted surgery is now a common practice for several surgical operations and will probably become the most exploited surgical procedure of the future (Song et al., 2009; Parisi et al., 2017; Kulaylat et al., 2018; Sheetz et al., 2020). In this context, the da Vinci robot (Intuitive Surgical Inc.) demonstrated effective recovery of hand-eye coordination and motion dexterity. Research done in the last decade has been focused on overcoming existing deficiencies of robotic surgery systems, e.g., lack of haptic feedback (Okamura, 2009; Manganelli et al., 2010; Meli et al., 2014; Mohareri et al., 2014), enhancement of system integration (Handini et al., 2004; Leven et al., 2005; Takács et al., 2015), and combination of augmented reality navigation functionalities (Sugimoto et al., 2010; Yamamoto et al., 2012; Tabrizi and Mahvash, 2015). Other research and development activities aim to realize outstanding training systems, including the next generation of virtual reality simulators (Van der Meijden et al., 2010; Fontanelli et al., 2018).

More than one thousand publications are available in literature discussing medical robots control approaches and surgeon side console (Beasley, 2012). A bilateral control, with position-position architecture, is the standard approach to control short distance tele-robotic systems for surgery, according to Niemeyer and Slotine (1991). For instance, in setups equipped with the da Vinci robot the position of the surgeon’s hand is mapped into the position of the surgery tool and the foot-pedal enables a temporary disconnection between the two sides to reconfigure the control manipulator into a more comfortable and ergonomic pose. For some applications, impedance control and switched-impedance control are used as a valid alternative (Cheng and Tavakoli, 2018). For instance, a strategy exploiting force based model predictive control for beating heart surgery is presented by Bebek and Cavusoglu (2006). These approaches are usually reinforced by identification techniques of robotic arms as described in Fontanelli et al. (2017), and by techniques of shared autonomy as motion compensation, collision avoidance (Moccia et al., 2020), active constrains, and shared task space (Bowyer et al., 2013).

An innovative surgery control console is presented in Sukhoon et al. (2009), the developed robot controller fully replicate the reference movements. Similarly, in Kim et al. (2013) the authors propose a control device mechanically similar to the robot end effector, coupled with a system for regulating the joints stiffness in accordance with the exerted force. On this line, new consoles are designed to recover tactile perception integrating haptic technologies. For instance, (Yin et al., 2015) used magnetorheological fluids to return haptic sensations. Grounded commercial haptic devices as Omega (Force Dimension, CH) or Falcon (Novint Technologies Inc., United States) replace the control side in some experiments to project a more portable and sensorily complete experience of control as in the case reported in Tang et al. (2016). In Madhani et al. (1998) a solution built with two PHANToM haptic devices is presented, whereas (Cavusoglu et al., 1999) shows a six DoFs laparoscopic telesurgery workstation composed of an Immersion System Impulse Engine 3000 and two additional motors. the authors in Hu et al. (2016) developed a tendon driven exoskeleton to control a surgical robot and provide force stimuli, a camera system tracks the positions of fingers of the surgeon, while bilateral Bowden cables return kinesthetic information.

**FIGURE 1:**
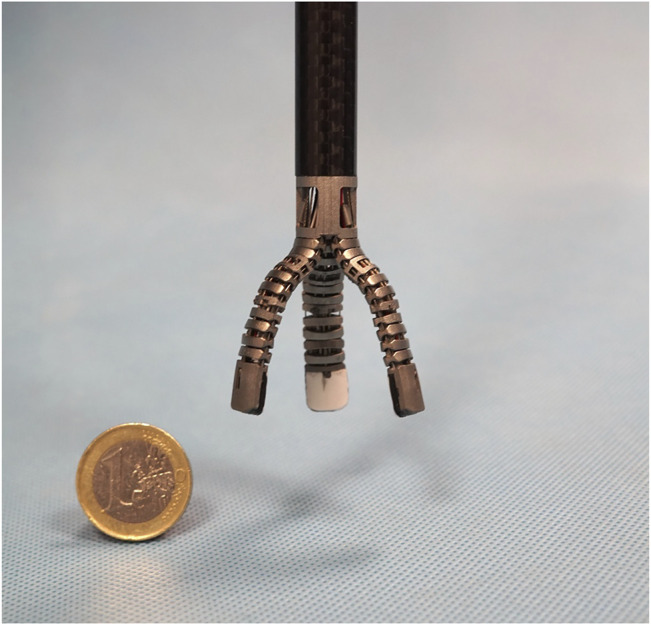
The MUSHA hand.

However, an open issue is the limited number of DoFs of robotic surgical end-effectors. In fact, the surgeon side manipulators of the da Vinci robot provides only eight DoFs to control the (Patient Side Manipulator) PSM position and the orientation and enclosure state of the common EndoWrist (Intuitive Surgical Inc., United States), bipolar and monopolar tools used to grasp, stamp or cut the organic tissues. Introducing extra degrees of freedom requires the development of suitable control (Gioioso et al., 2013; Meeker et al., 2018), or the development of alternative control consoles to move them independently. For instance, the MUSHA hand (depicted in Figure 1) is a three-soft-finger robotic gripper for surgical application (Liu et al., 2020). It is developed to be connected with the da Vinci robot, but it has more degrees of actuation (DoA) than DoFs of surgeon console interface. For this reason, the DoAs of the MUSHA hand are controlled synergistically, however, this reduces the potential of the instrument by limiting the number of configurations that can be reached by the fingers.

The contribution of this paper is a human to MUSHA hand mapping algorithm to perform full teleoperation of surgical hand DoAs, without increasing the mechanical complexity of the surgeon console. For this purpose, we use a camera system to track the human hand motion. The algorithm can easily be adapted to any robotic tool, the resulted algorithm is corroborated by a theoretical analysis of stability and it is followed by a preliminary study about forces exerted by fingertips.

## 2 Materials and Methods

The teleoperated MUSHA hand system is composed of surgical gripper and the da Vinci robot arm in patient side, while the surgeon side is comprehensive of a human hand tracker system. Communication between the surgeon and patient side is provided by Robot Operating System (ROS).

### 2.1 MUSHA Hand Modelling

**FIGURE 2:**
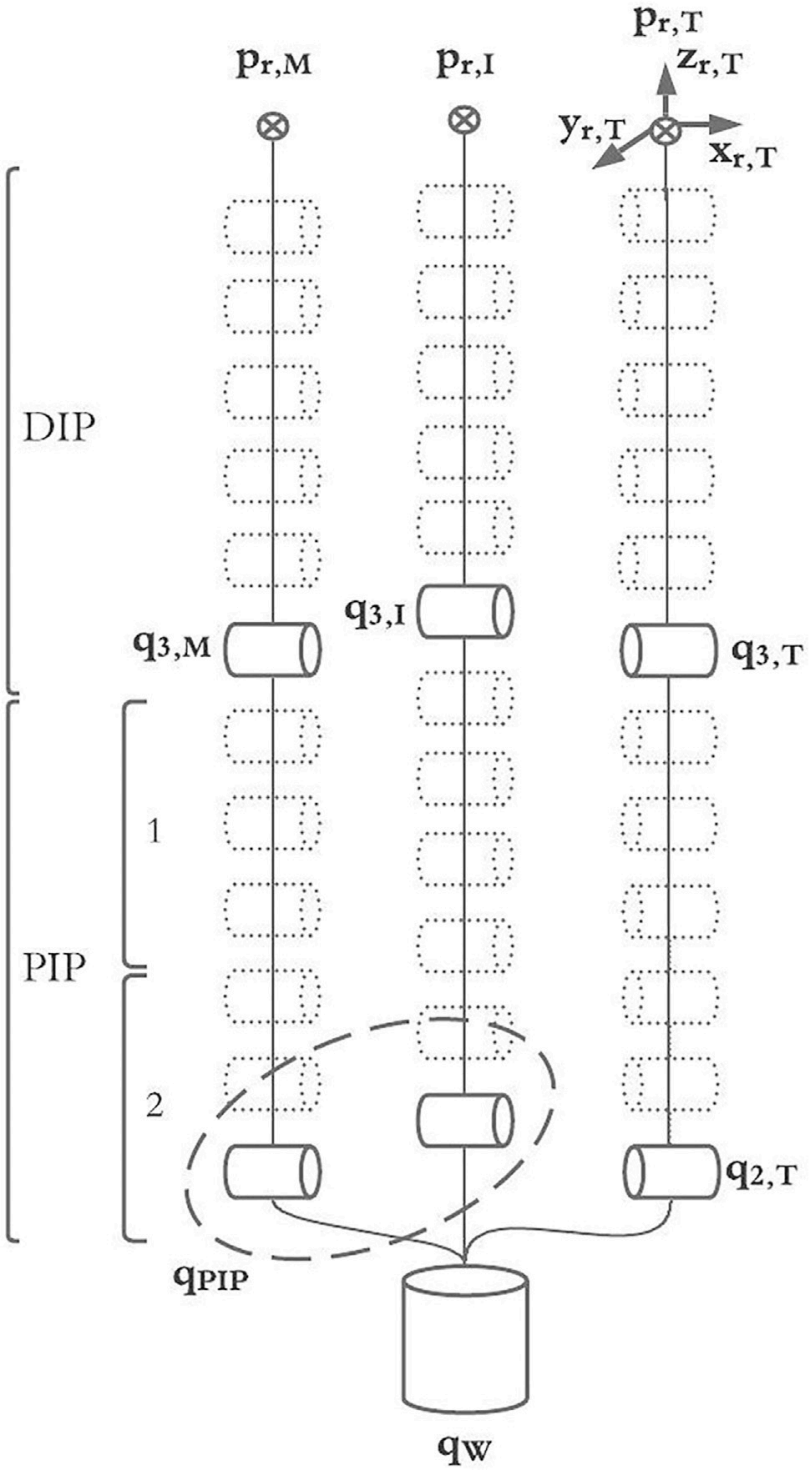
The full kinematic model of the MUSHA hand and the reduced model into joint actuated space.

**FIGURE 3:**
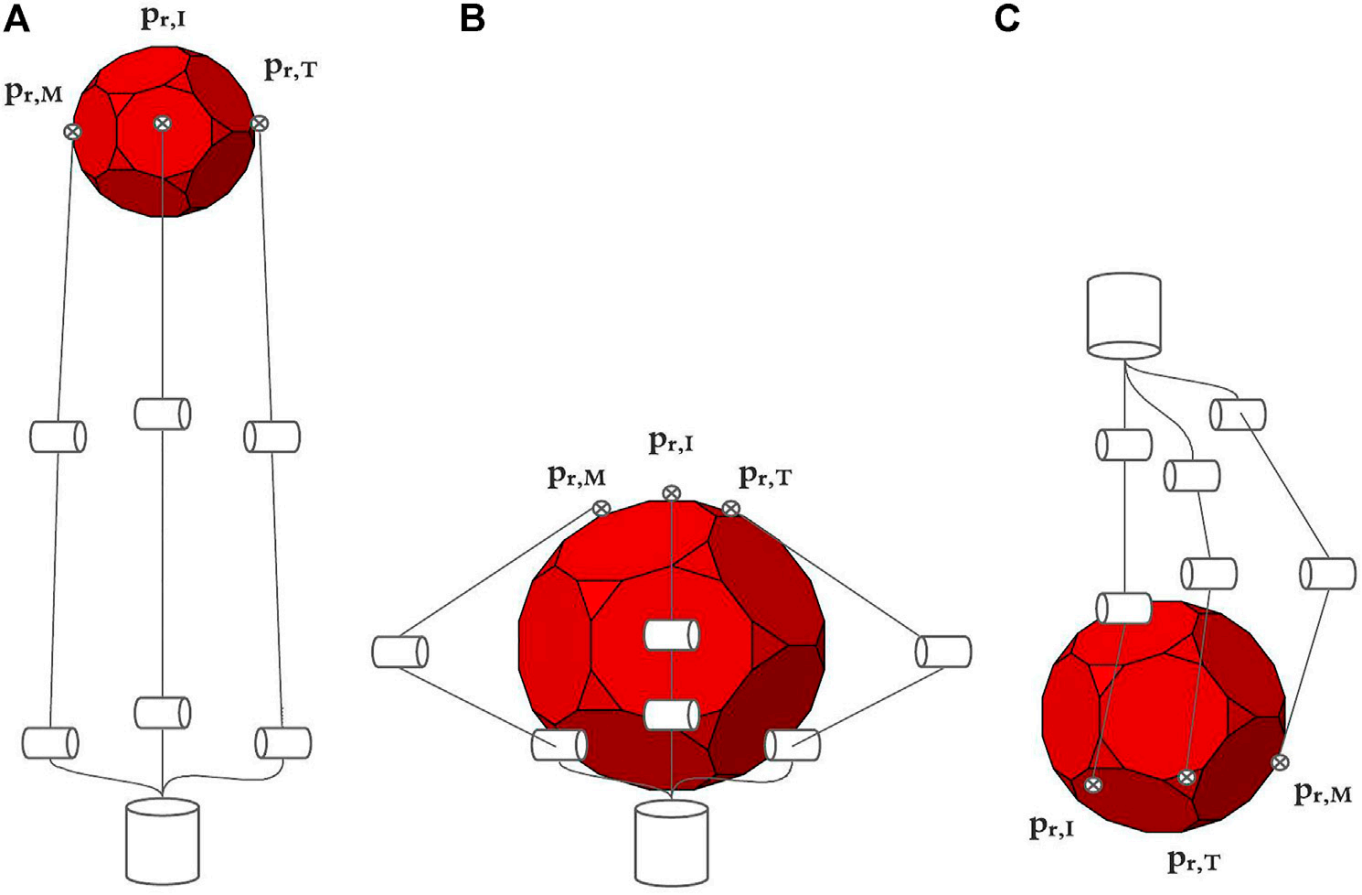
Main grasping method of MUSHA, from left to right are respectively depicted the approach poses in case of precision grasp (Pr) in A, power grasp (Po) in B and retractor (Rt) in C.

The MUSHA hand is the main and innovative part of the teleoperation system, while the PSM of the da Vinci robot is already a known system. Then, in what follows we describe the multi-functionality of the MUSHA hand and its kinematic model which is crucial in the development of the entire control approach. According to Liu et al. (2019), and as anticipated in Section 1, the tool is a three-fingered underactuated miniature hand, each finger is composed of 12 segments and connected with a one DoF wrist, the kinematic model is composed of 37 joints connected by 36 links. An optoelectronic force sensor is designed, calibrated and presented in Liu et al. (2020), and located at the tip of each finger. In the range of the sensors, equal to 4 N, the maximal error on x-, y-, and z-direction is 0.28, 0.24, and 0.75 N, respectively. The MUSHA is actuated by the four motors of da Vinci PSM plus two additional motors. As a result, six joints are independent and directly connected to actuators by tendons, while the other ones are passive. For these reasons, it is possible to define a reduced kinematic model composed of actuated joints and equivalent links (Figure 2). The kinematic structure and the actuation system are designed to be multi-functional allowing the MUSHA hand performing three main methods of grasp, depicted in Figure 3: 1) power grasp (*Po*), 2) precision grasp (*Pr*) and 3) retractor (*Rt*). During the power grasp method, all fingertips and phalanges are involved to hold firmly tissue, in the precision grasp method only the fingertips are used to pinch tissue, and the retractor method allows the retraction of the tissues that come in contact with phalanges of index and middle fingertips. The relationship between joints and the tips position for a single robotic finger results as:

$$\begin{array}{cc} & x_{r,\rho }=\cos\left(q_{1,\rho }\right)\left(a_{2}\cos\left(q_{2,\rho }\right)+a_{3}\cos\left(q_{2,\rho }+q_{3,\rho }\right)\right)\\ & y_{r,\rho }=\sin\left(q_{1,\rho }\right)\left(a_{2}\cos\left(q_{2,\rho }\right)+a_{3}\cos\left(q_{2,\rho }+q_{3,\rho }\right)\right)\\ & z_{r,\rho }=a_{2}\sin\left(q_{2,\rho }\right)+a_{3}\sin\left(q_{2,\rho }+q_{3,\rho }\right)\\ & \forall \rho \in \left\{T,I,M\right\} \end{array}$$ (1)

where *q*_*i*,*ρ*_ is the value of the *i-th* joint of the robotic hand *r* *ρ* *-th* finger and *a*_*i*_ is the length of the *i-th* link. We refer with *T, I, M* the thumb, index, and middle finger, respectively. Thanks to the mechanical properties of the robotic tool tendon-driven transmission, *q*_3,*ρ*_ and *q*_2,*ρ*_ are decoupled and thus they can be independently controlled, it follows that the model can be rewritten as

$$\begin{array}{cc} & x_{r,\rho }=\cos\left(q_{1,\rho }\right)\left(a_{2}\cos\left(q_{2,\rho }\right)+a_{3}\cos\left(q_{3,\rho }\right)\right)\\ & y_{r,\rho }=\sin\left(q_{1,\rho }\right)\left(a_{2}\cos\left(q_{2,\rho }\right)+a_{3}\cos\left(q_{3,\rho }\right)\right)\\ & z_{r,\rho }=a_{2}\sin\left(q_{2,\rho }\right)+a_{3}\sin\left(q_{3,\rho }\right)\\ & \forall \rho \in \left\{T,\;I,\;M\right\}. \end{array}$$ (2)

**FIGURE 4:**
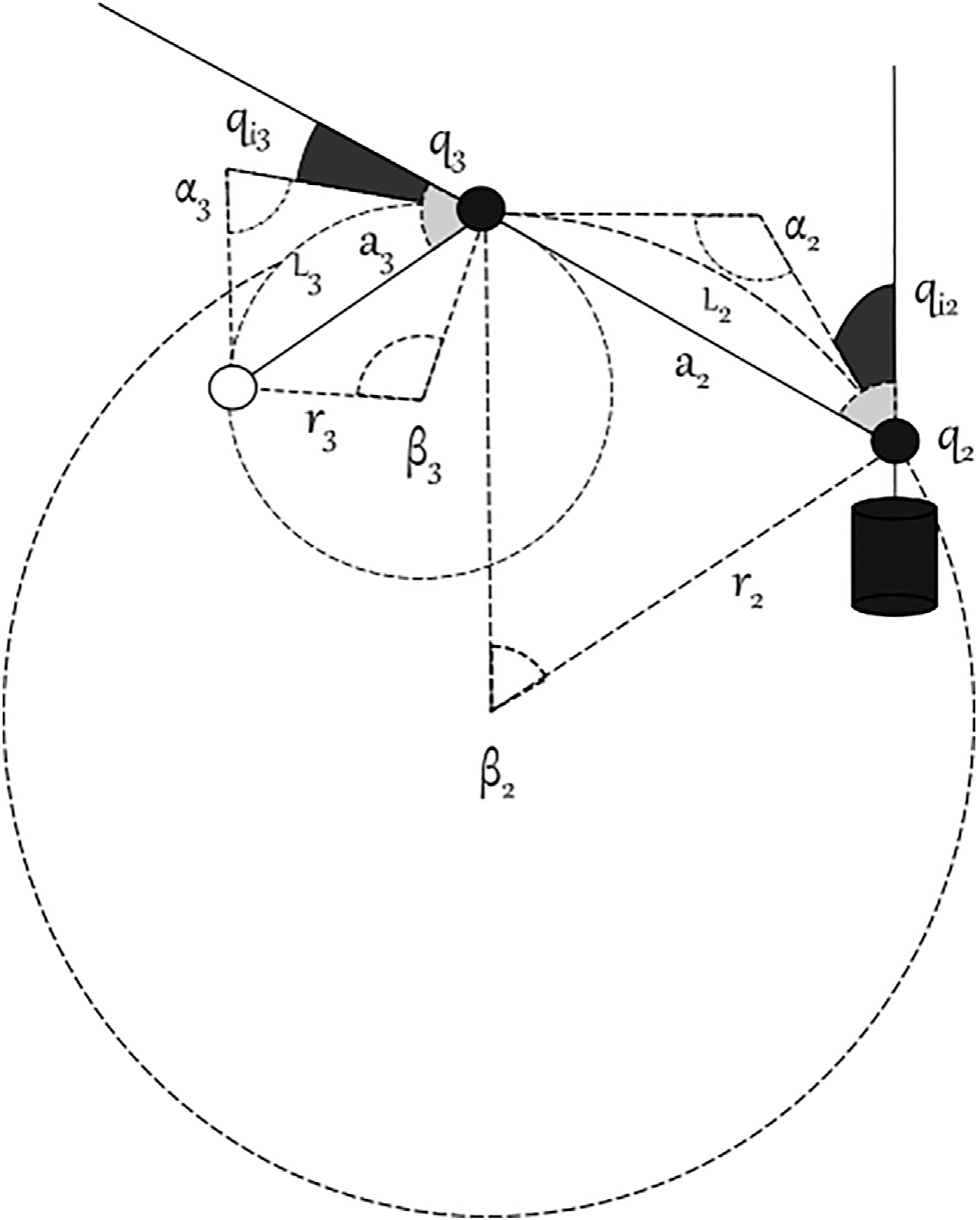
Details of a bended MUSHA finger.

The complete kinematic model of the MUSHA hand, *p*_*r*_ = *K*(⋅), is obtained exploiting (2) for each finger, thus *p*_*r*_ = *K*(*q*_1,*T*_, *q*_2,*T*_, *q*_3,*T*_, *q*_1,*I*_, *q*_2,*I*_, *q*_3,*I*_, *q*_1,*M*_, *q*_2,*M*_, *q*_3,*M*_). According to the sub-actuation constrains we define the wrist angle position, *q*_*W*_, and the index and medium proximal one, *qPIP*,:

$$\left\{\begin{array}{c} q_{W}\;=q_{1,I}\;\\ q_{W}\;=q_{1,T}+\frac{2\pi }{3}\;\\ q_{W}\;=q_{1,M}-\frac{2\pi }{3}\; \end{array}\right.\text{ and }\left\{\begin{array}{c} q_{PIP}\;=q_{2,M}\;\\ q_{PIP}\;=q_{2,I}\; \end{array}\right.$$ (3)

we can simplify the kinematic relationship, obtaining:

$$p_{r}=K\left(q_{W},q_{2,T},q_{3,T},q_{PIP},q_{3,I},q_{3,M}\right)$$ (4)

where *p*_*r*_ is a vector containing the fingertips position expressed in Cartesian coordinate. It is worth noticing that in the exploited model, the length of the links *a*_2_ and *a*_3_ depends on the joint values. Indeed, the coupling motion of the phalanx segments (generated by the tendons) causes links shrinkage. In accordance with the chord theorem (see Figure 4), the length of the links can be computed as

$$\begin{array}{c} a_{i,\rho }=2r_{i,\rho }\sin\left(\frac{\beta_{i}}{2}\right). \end{array}$$ (5)

Let $q_{i_{j},\rho }$ be the joint *j* in the phalanx *i* of the finger *ρ*, ∀*i* ∈ {2, 3} and ∀*ρ* ∈ {*T*, *I*, *M*}. Consequently, it is possible to write the length of the chord in kinematic model and the length of the link in the actuated model as follows:

$$a_{i,\rho }=\left\{\begin{array}{c} \left(\frac{2L_{i,\rho }}{\sum_{j=2}^{6}q_{i_{j},\rho }+\sum_{j=1}^{5}q_{i_{j},\rho }}\right)\sin\left(\frac{\sum_{j=2}^{6}q_{i_{j},\rho }+\sum_{j=1}^{5}q_{i_{j},\rho }}{2}\right)\;\\ L_{i,\rho }\text{ if }q_{i,\rho }=0\; \end{array}\right.$$ (6)

where *L*_*i*_, *ρ* is the length of the arc of the *i-th* phalanx of the finger *ρ*. Assuming that the action of the tendons is equal on all the segments, the angular displacements are equal to a fraction of the equivalent joint in the reduced model:

$$q_{i_{j},\rho }=\frac{q_{i,\rho }}{6}\;\forall j\in \left[1,6\right]\text{, }\forall i\in \left\{2,3\right\}\text{ and }\forall \rho \in \left\{T,I,M\right\}.$$ (7)

As a consequence, ∀*ρ* ∈ {*T*, *I*, *M*} the single finger model becomes:

$$x_{r,\rho }=\cos\left(q_{1,\rho }\right)\left(a_{2,\rho }\cos\left(q_{2,\rho }\right)+a_{3,\rho }\cos\left(q_{3,\rho }\right)\right)$$ (8)

$$y_{r,\rho }=\sin\left(q_{1,\rho }\right)\left(a_{2,\rho }\cos\left(q_{2,\rho }\right)+a_{3,\rho }\cos\left(q_{3,\rho }\right)\right)$$

$$z_{r,\rho }=a_{2,\rho }\sin\left(q_{2,\rho }\right)+a_{3,\rho }\sin\left(q_{3,\rho }\right)$$

$$a_{2,\rho }=\left\{\begin{array}{c} \left(\frac{6L_{2,\rho }}{5q_{2,\rho }}\right)\sin\left(\frac{5q_{2,\rho }}{6}\right)\;\\ L_{2,\rho }\text{ if }q_{2,\rho }=0\; \end{array}\right.$$

$$a_{3,\rho }=\left\{\begin{array}{c} \left(\frac{6L_{3,\rho }}{5q_{3,\rho }}\right)\sin\left(\frac{5q_{3,\rho }}{6}\right)\;\\ L_{3,\rho }\text{ if }q_{3,\rho }=0\; \end{array}\right.$$

Therefore, the explicit full hand model (reported in the Supplementary Material) has 14 equations nine of which linearly independent, in six variables. Finally, we can express ${\dot{p}}_{r}=J_{r}^{w}{\dot{q}}_{r}$ , where $J_{r}^{w}$ is the Jacobian matrix of the robotic fingers of MUSHA expressed in reference frame of the wrist. The differential model is fully reported in the Supplementary (see Supplementary Section S5.2).

### 2.2 Human Hand Modelling

**FIGURE 5:**
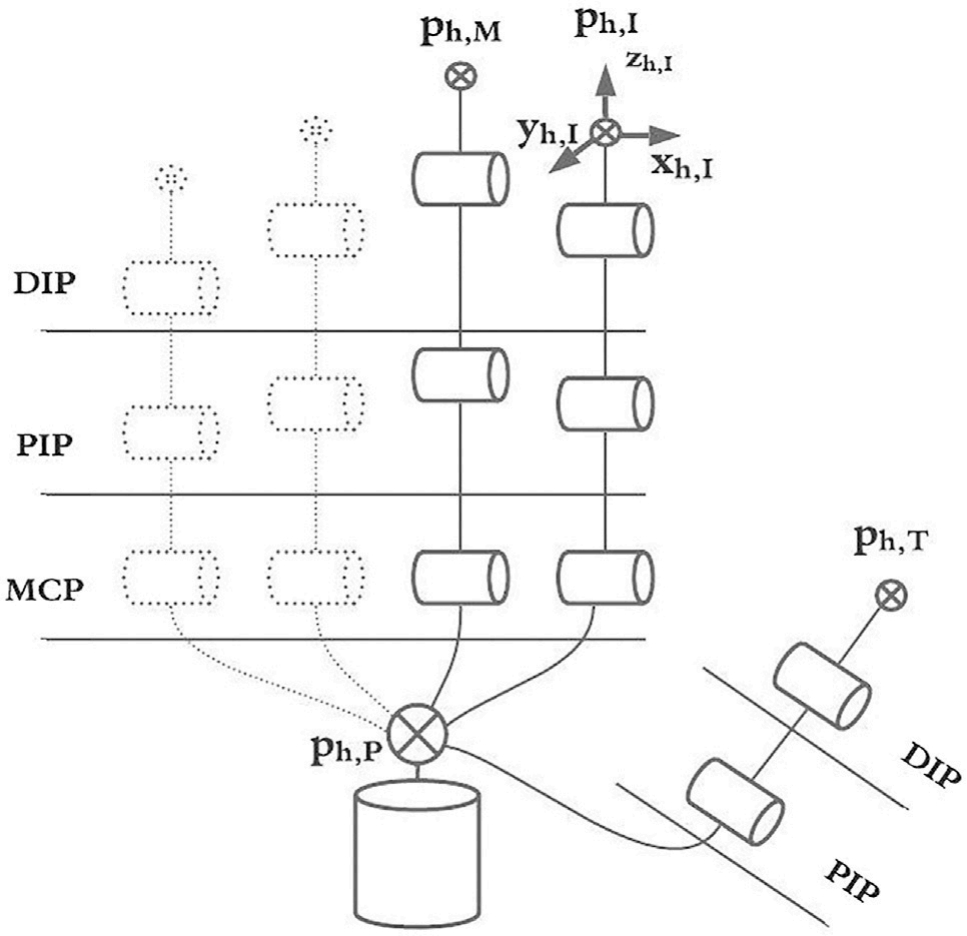
The simplified kinematic model of the human hand, structure with links, joints, base, and rotation axes are in evidence.

In this section, we report the simplified kinematic model of the hand, which will be instrumental in the design of the control technique. Even if a complete human hand model has about 30 DoF (Lee and Kunii, 1995), for the sake of simplicity and without loss of generality we use a simplified kinematic hand structure. We modeled each finger as a planar kinematic chain, with three 1-D hinges. As in Lisini et al. (2017), we assume that each finger has the metacarpal (MC) bone fixed with respect to the hand frame, and it is characterized by three DoFs. In a more complete model of the human hand, the thumb has at least five DoFs: two in the trapeziometacarpal (TM) joint, two in the metacarpophalangeal (MCP) joint, and one in the interphalangeal (IP) joint. However, the thumb abduction/adduction motion range of the MCP joint usually can be neglected so the thumb can be modeled with two DoFs. Index, middle, ring, and pinky fingers have one DoFs in the MCP revolute joint for flexion/extension, one in the proximal IP (PIP), and one in the distal IP (DIP). Figure 5 shows the model of the hand used in this work. Therefore, the exploited hand model has 20 DoFs. i.e., 14 DoFs describing the fingers and six DoFs (position and orientation) for the palm. Finally, we introduce the relationship between fingertips and joints velocities, which can be described by the differential model:

$${\dot{\hat{p}}}_{h}=J_{h}\left(q_{h}\right){\dot{q}}_{h}$$ (9)

where the matrix $J_{h}\in R^{15\times 20}$ is the hand Jacobian, that maps the joint velocities ( ${\dot{q}}_{h}\in R^{20}$ ) to the Cartesian fingertip velocities ( ${\dot{\hat{p}}}_{h}\in R^{15}$ ).

### 2.3 Mapping Algorithms to Teleoperate da Vinci Arm and MUSHA Hand

Starting from the definition of the human hand and the MUSHA hand models, the developed method to control the MUSHA hand and the da Vinci robot arm are explained. A standard point-to-point mapping technique is used to control the PSM, while an innovative approach based on the theory of mapping in object domain to teleoperate the MUSHA fingers is designed and implemented. We assume that the human and the robotic hands are constantly in touch with a similar virtual object, all possible contact points are called *active points*. The positions of MUSHA active points *p*_*r*_ are defined as the positions of fingertips, while the human active points ( $p_{h}\subset {\hat{p}}_{h}$ ) are given by the positions of palm (P), thumb, index, and middle fingertips:

$$p_{h}={\left[p_{h,P},p_{h,T},p_{h,I},p_{h,M}\right]}^{T}.$$ (10)

We indicate with *p*_*h*,*ρ*_ a vector of Cartesian positions of the *ρ*-*th* finger of the human hand *h* expressed in the world reference frame:

$$p_{h,\rho }={\left[x_{h,\rho },y_{h,\rho },z_{h,\rho }\right]}^{T},\;\forall \rho \in \left\{P,T,I,M\right\}.$$ (11)

Depending on the grasping method, we define the vector of *interaction points*, *c*_*h*_, equal to a subset of active points of the human hand involved in the desired grasp, and evaluated as:

$$c_{h}=\int_{t}{\dot{c}}_{h}\left(\tau \right)d\tau$$ (12)

being

$${\dot{c}}_{h}=I\Omega {\dot{p}}_{h}\text{ with }I\in R^{12\times 12}$$ (13)

and

$$\Omega =\left\{\begin{array}{cc} 1_{12\times 1}\; & \text{if method}=\mathrm{Pr}\\ {\left(0_{1\times 3},1_{1\times 9}\right)}^{T}\; & \text{if method}=Po\\ {\left(1_{1\times 3},0_{1\times 3},1_{1\times 6}\right)}^{T}\; & \text{if method}=Rt \end{array}\right.$$ (14)

It is worth noticing that Ω is a selection vector that is evaluated on-line to allow switch control reference without discontinuity and according to the grasping method required. The palm-thumb distance is used to recognize the current grasping method.

The reference of PSM in cartesian space is calculated as integration over time *τ* of the velocity components of the user hand centroid ${\dot{\tilde{c}}}_{h}$ until the time instant *t*. In this way, during a temporary disconnection between the patient side and control side, the relocation of the palm in more ergonomic poses or in a position far from the boundaries of the vision cone of the camera system is allowed. This results in:

$$p_{PSM}\left(t\right)=p_{PSM}\left(t-\tau \right)+\int_{t}{\dot{\tilde{c}}}_{h}\left(\tau \right)d\tau$$ (15)

Where the value of centroid changes according to method of grasping, in fact:

$${\tilde{c}}_{h}=\frac{\underset{\rho \in \left\{P,T,I,M\right\}}{\sum }\left(c_{h,\rho }\right)}{N}\text{ with }N=\left\{\begin{array}{c} 3\text{ if method}\neq Po\\ 4\text{ if method}=Po \end{array}\right.$$ (16)

Similarly, the orientation of the human hand is exploited to orient the PSM. To this end Euler angles in roll (*α*), pitch (*β*), and yaw (*γ*) convention are computed as:

$$\alpha =\frac{1}{2}\tan^{-1}\left(\frac{2\mu_{0,1,1}}{\mu_{0,2,0}-\mu_{0,0,2}}\right),$$ (17)

$$\beta =\frac{1}{2}\tan^{-1}\left(\frac{2\mu_{1,0,1}}{\mu_{0,0,2}-\mu_{2,0,0}}\right),$$ (18)

$$\gamma =\frac{1}{2}\tan^{-1}\left(\frac{2\mu_{1,1,0}}{\mu_{2,0,0}-\mu_{0,2,0}}\right),$$ (19)

being *μ*_*i*,*j*,*k*_ the second order central moments of cloud of contact points

$$\mu_{i,j,k}=\underset{\rho \in P,I,T,M}{\sum }{\left(x_{h,\rho }-{\tilde{x}}_{h}\right)}^{j}{\left(y_{h,\rho }-{\tilde{y}}_{h}\right)}^{i}{\left(z_{h,\rho }-{\tilde{z}}_{h}\right)}^{k}$$ (21)

We exploited a sphere as virtual object to easily project the human hand motions into MUSHA fingers and wrist movements.

The roto-translations and deformations of a sphere can be completely defined by the following seven parameters: 1) $o_{h}\in R^{3}$ Cartesian coordinate of the center of sphere; 2) $\theta_{h}\in R^{3}$ the orientation of the frame attached to the sphere with respect to the world frame, expressed as roll, pitch, and yaw angles; 3) $r_{h}\in R$ the sphere radius. Combining (13) and the aforementioned seven parameters, it is possible to obtain a linear relationship between human hand motions and sphere transformation

$${\dot{c}}_{h,\rho }={\dot{o}}_{h}+{\dot{\theta }}_{h}\times \left(c_{h,\rho }-o_{h}\right)+{\dot{r}}_{h}\left(c_{h,\rho }-o_{h}\right).$$ (22)

Thus, there exists a Jacobian matrix *J*_*hs*_ such that its pseudo-inverse, $J_{hs}^{†}$ , projects the human fingertip velocities into derivative parameters of the sphere:

$$\left[\begin{array}{c} \dot{o_{h}}\\ \dot{\theta_{h}}\\ \dot{r_{h}} \end{array}\right]=J_{hs}^{†}\left(o_{h},c_{h}\right)I\Omega \dot{p_{h}}$$ (23)

The resulting Jacobian matrix depends on the current position of interaction points of human hand and on the sphere center that is a function of the active points of the human hand. Indeed, the sphere center is the circumcenter of a triangle with three of the current interaction points as vertices.

$$o_{h}=c_{h,i}+\frac{{\left|\left(\Delta_{h,ki}\right)\right|}^{2}\left[\left(\Delta_{h,ji}\right)\times \left(\Delta_{h,ki}\right)\right]\times \left(\Delta_{h,ji}\right)}{2{\left|\left(\Delta_{h,ji}\right)\times \left(\Delta_{h,ki}\right)\right|}^{2}}+\frac{{\left|\left(\Delta_{h,ji}\right)\right|}^{2}\left(\Delta_{h,ki}\right)\times \left[\Delta_{h,ji}\left.\right)\times \left(\Delta_{h,ki}\right)\right]}{2{\left|\left(\Delta_{h,ji}\right)\times \left(\Delta_{h,ki}\right)\right|}^{2}}$$ (24)

where Δ_*h*,*ki*_ is the difference between the position vector of the *k-th* fingertip *c*_*h*,*k*_ in contact with the sphere and the *i-th* fingertip *c*_*h*,*i*_, then, (23) can be rewritten as:

$$\left[\begin{array}{c} \dot{o_{h}}\\ \dot{\theta_{h}}\\ \dot{r_{h}} \end{array}\right]=J_{hs}^{†}\left(p_{h}\right)I\Omega \dot{p_{h}}$$ (25)

As a further step towards the mapping, the sphere manipulated by the human is properly transformed into the one manipulated by the MUSHA through a scale factor *K*_*s*_, that is equal to the ratio between the radius of human and MUSHA manipulated spheres. The algorithm computes the Jacobian of the sphere manipulated by the robot (i.e., *J*_*rs*_) and evaluates the velocities of the robotic fingertips,

$${\dot{p}}_{r}=J_{rs}\left(p_{r}\right)K_{s}J_{hs}^{†}\left(p_{h}\right)I\Omega \dot{p_{h}}.$$ (26)

**FIGURE 6:**
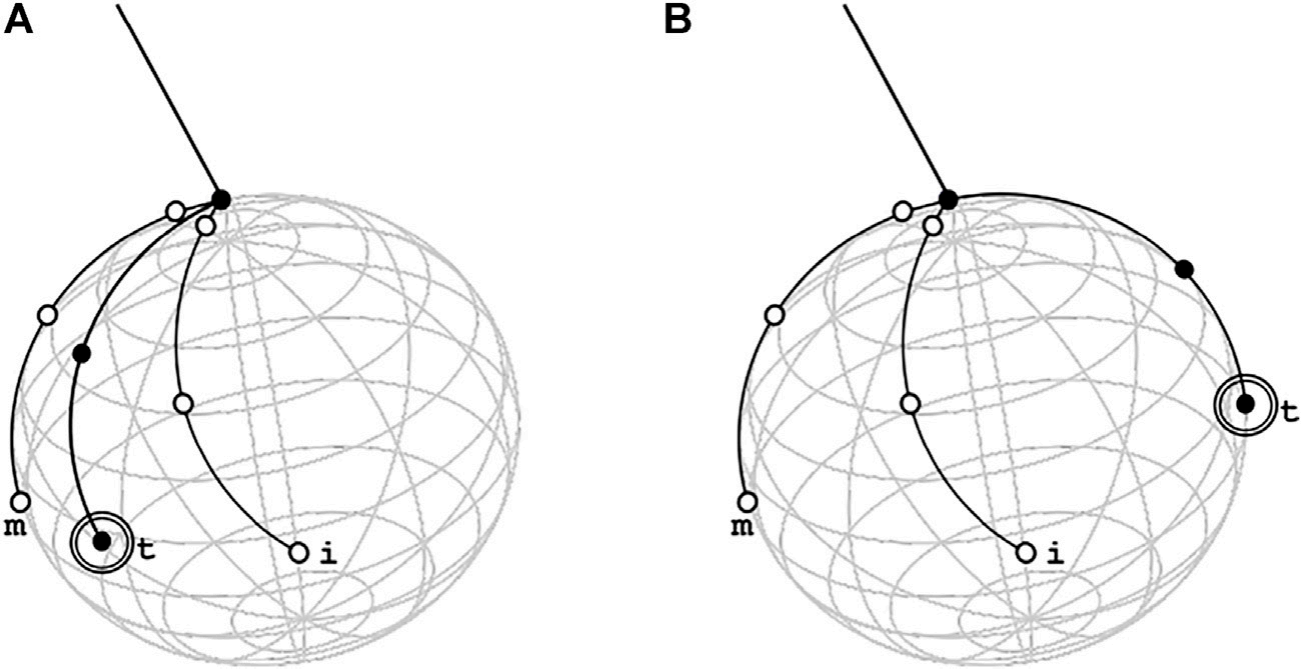
Graphic representation of thumb indeterminacy in retractor method with two reachable positions, in the correct one in **(A)** the thumb is between middle and index to allow the hand to reach the position scoop-like while in wrong one, depicted in **(B)**, the thumb is in opposition respect the other two fingers and the reached posed of MUSHA hand is equal to approach pose in power grasp method. This is a consequence of the high similarity between the virtual spheres generated by the contact points in the Rt case and in the Po case.

Velocities are estimated by a closed loop kinematic inversion

$${\dot{q}}_{r}=J_{r}^{w†}\left[\left(q_{r}\right)J_{rs}\left(p_{r}\right)K_{s}J_{hs}^{†}\left(p_{h}\right)I\Omega \dot{p_{h}}+K_{c}\left({\tilde{p}}_{r}-p_{r}\right)\right]$$ (27)

where $J_{r}^{w}$ , *J*_*rs*_ and *J*_*hs*_ are the Jacobian matrices of the robot, of the sphere manipulated by the robot, and of the sphere manipulated by the user, respectively. $K_{c}\left({\tilde{p}}_{r}-p_{r}\right)$ is the corrective action on the error between the current position of MUSHA interaction points *p*_*r*_ and the desired ones ${\tilde{p}}_{r}$ , weighted by the gain *K*_*c*_. It is worth pointing out that the pseudo-inversion of *J*_*hs*_ induces an indeterminacy in the estimated positions far from the current pose in case of multiple solutions, for this reason, the right position of the thumb in the retractor method may be not correctly computed. This situation is visually depicted in Figure 6. A projection into the null-space of mapping actions is used to overcome this limitation. To this end, (27) is extended as follows,

$${\dot{q}}_{r}=J_{r}^{w†}\left(q_{r}\right)\left[J_{rs}\left(p_{r}\right)K_{s}J_{hs}^{†}\left(p_{h}\right)I\Omega \dot{p_{h}}+K_{c}\left({\tilde{p}}_{r}-p_{r}\right)+J_{r}^{w}\left[I_{6\times 6}-{\left(J_{rs}K_{s}J_{hs}I\Omega \right)}^{†}\left(J_{rs}K_{s}J_{hs}I\Omega \right)\right]u\right]$$ (28)

where

$$u=\left\{\begin{array}{cc} 0_{6\times 1} & \text{if method}\neq Rt\\ K_{Rt}\left(q_{Rt}-q_{r}\right) & \text{if method}=Rt \end{array}\right.$$ (29)

being *K*_*Rt*_ the gain that multiplies the error between current joint position *q*_*r*_ and *q*_*Rt*_, the expected joint position in retractor pose. Regarding the initial condition, at the beginning of any task, the MUSHA lies in a rest position with all the fingers closed with joint configuration $q_{r0}=0_{6\times 1}$ . This particular configuration results in a kinematic singularity, in fact when *q*(*t*) = *q*_*r*0_ the rank of Jacobian of robot decreases, $Rank\left(J_{r}^{w}\right)<6$ . For this reason, an initialization procedure is required. In accordance with

$$q_{r_{0}}=\left\{\begin{array}{c} \arg\left(\max_{q}\left(\sqrt{\det\left(J_{r}^{w}\left(q\right)J_{r}^{wT}\left(q\right)\right)}\right)\right)\\ q_{i,\rho }\in \left[q_{i,\rho_{\min}},q_{i,\rho_{\max}}\right]\;\forall i\text{, }\forall \rho \end{array}\right.$$ (30)

we select $q_{r_{0}}=\left[0,\frac{\pi }{9},-\frac{\pi }{9},\frac{\pi }{9},-\frac{\pi }{9},-\frac{\pi }{9}\right]$ as initial position. It corresponds to the joint configuration with the maximum manipulability. In conclusion, combining (28–30), we obtain the entire human-MUSHA hand joints mapping:

$$q_{r}=\left\{\begin{array}{cc} q_{r_{0}} & \text{ if }t=0\\ \int_{t}{\dot{q}}_{r}\left(\tau \right)d\tau & \text{ if }t\neq 0 \end{array}\right.$$ (31)

with

$${\dot{q}}_{r}=J_{r}^{w†}\left(q_{r}\right)\left[J_{rs}\left(p_{r}\right)K_{s}J_{hs}^{†}\left(p_{h}\right)I\Omega \dot{p_{h}}+K_{c}\left({\tilde{p}}_{r}-p_{r}\right)+J_{r}^{w}\left[I-{\left(J_{rs}K_{s}J_{hs}I\Omega \right)}^{†}\left(J_{rs}K_{s}J_{hs}I\Omega \right)\right]u\right]$$ (32)

### 2.4 User Interface, Software and Communication

The camera system LeapMotion (Ultraleap inc., United States) is used to acquire active points of human hand. This camera system is composed of two monochromatic IR cameras and three LEDs that observe a hemispherical area and capture snapshots of up to 200 frames per second, and it returns information about positions of some fixed notable points of acquired hand as tips of fingers, the center of palm and knuckles. A ROS node acquires these from LeapMotion and publishes the values of active points, selecting only those useful for the mapping algorithm. To test the control law a virtual model of MUSHA hand is imported in V-rep (Coppelia Robotics GmbH, CH), and here its kinematic and dynamic behavior is simulated.

## 3 Results

First of all, a theoretical analysis of applied forces is conducted to evaluate the effect of contact between MUSHA and a generic organic tissue for each method of grasp, then a validation of the control algorithm is pursued to demonstrate its stability. An experimental campaign follows, during which naive participants are asked to complete a pick and place operation using the virtual model of the MUSHA hand.

### 3.1 Theoretical Grasping Forces Evaluation

During surgery, the grasping and manipulation of tissue may generate both internal and external forces. The former impresses deformation and allows to avoid the sliding of the manipulated tissue, the latter generates twists. In accordance with the grasping theory (Prattichizzo and Trinkle, 2016), it is possible to evaluate from the applied force (i.e., *λ*) the components that generate the squeezing (i.e., *ξ*), and the ones that generate twisting (i.e., *ω*). This can be done by exploiting the grasping matrix $G\in R^{6\times 18}$ and the null space projector $N_{G}\in R^{18\times 18}$ , following the relationship

$$\lambda =-G^{†}\omega +N_{G}\xi$$ (33)

with

$$G^{T}=H{\tilde{G}}^{T}.$$ (34)

**FIGURE 7:**
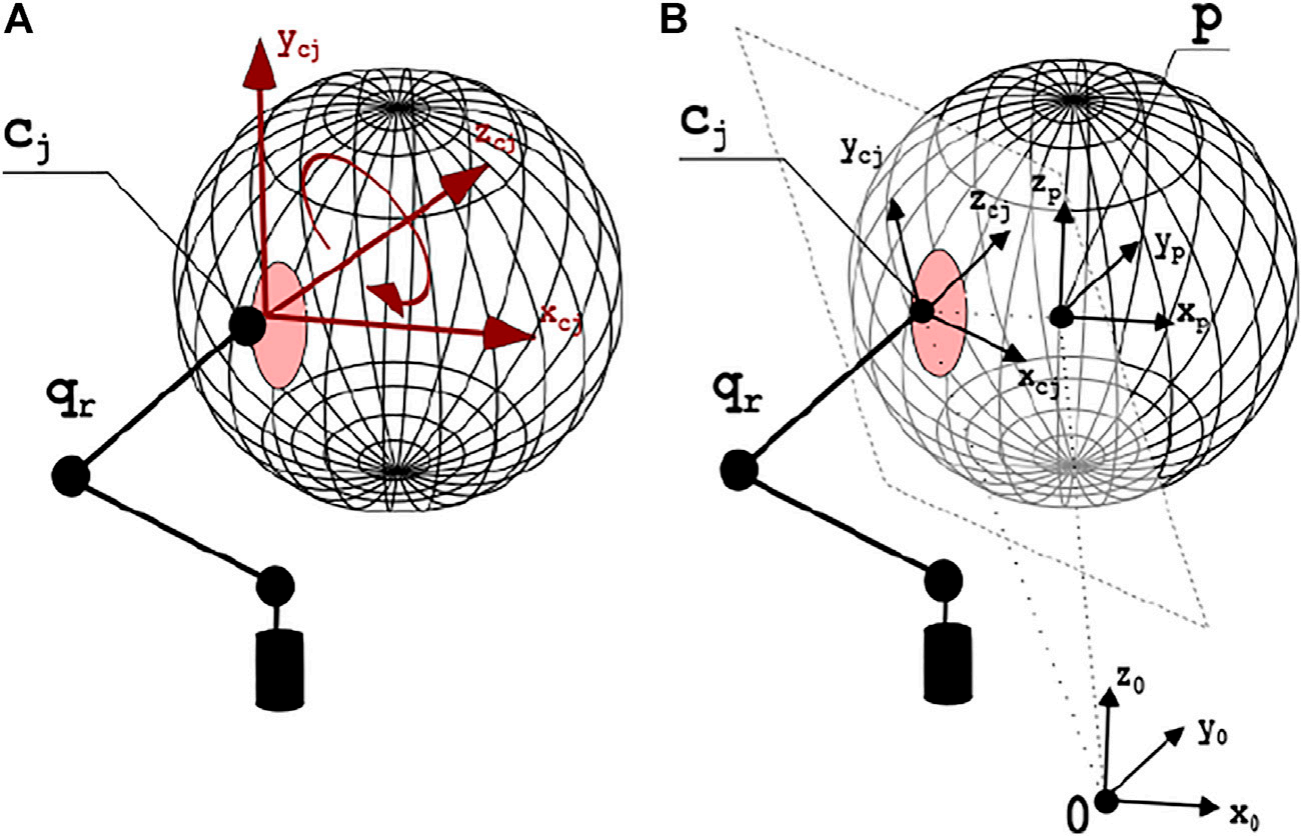
In **(A)**, the contact model with one finger. The red area surrounding the contact point *c*_*j*_ is the deformation zone. Red arrows display the directions in which motion is not allowed, thus it is possible to apply force or torques. In **(B)**, the contact surface and the axes of the main reference systems are depicted.

${\tilde{G}}^{T}$ is the transposed grasping matrix that maps sources of twist from contact point reference to world reference. *H* is a selection matrix depending on the contact finger model. Considering the consistency of organic tissues, it is possible to approximate the grasp with the *Soft Finger* (SF) contact model. Interested reader is referred to Prattichizzo and Trinkle (2016) for further details. Therefore, admitting the applicability of the principle of kineto-static duality, it is possible to apply generalized forces exclusively in the directions of constrained motions. Directions of force application are shown in Figure 7 in case of contact with one finger. We assumed SF contact model for each point, thus the selection matrix *H* can be defined as

$$H=\left[\begin{array}{cccc} H_{T} & 0 & 0\\ 0 & H_{I} & 0\\ 0 & 0 & H_{M} & \end{array}\right]\in R^{18\times 18}$$ (35)

with

$$H_{T}=H_{I}=H_{M}=\left[\begin{array}{cccccc} 1 & 0 & 0 & 0 & 0 & 0\\ 0 & 1 & 0 & 0 & 0 & 0\\ 0 & 0 & 1 & 0 & 0 & 0\\ 0 & 0 & 0 & 0 & 0 & 0\\ 0 & 0 & 0 & 0 & 0 & 0\\ 0 & 0 & 0 & 0 & 0 & 1 \end{array}\right].$$ (36)

Consequently, the complete grasp matrix *G* ^*T*^ can be modeled as $G^{T}=H{\tilde{G}}^{T}$ , being

$${\tilde{G}}^{T}=\left[\begin{array}{ccc} {\bar{R_{T}}}^{T} & 0 & 0\\ 0 & {\bar{R_{I}}}^{T} & 0\\ 0 & 0 & {\bar{R_{M}}}^{T} \end{array}\right]\left[\begin{array}{c} P_{T}^{T}\\ P_{I}^{T}\\ P_{M}^{T} \end{array}\right],\;{\tilde{G}}^{T}\in R^{18\times 6}$$ (37)

with

$${\bar{R}}_{j}^{T}=\left[\begin{array}{cc} R_{j}^{T} & 0\\ 0 & R_{j}^{T} \end{array}\right]\;\forall j\in \left\{T,I,M\right\},$$ (38)

and

$$P_{j}^{T}=\left[\begin{array}{cc} I & -S\left(c_{j}-p_{ts}\right)\\ 0 & I \end{array}\right]\;\forall j\in \left\{T,I,M\right\}$$ (39)

where *R* ^*T*^ ∈ *R* ^3×3^ are the rotation matrices describing the orientation of each contact point reference frame Σ_*j*_, *j* = *T*, *I*, *M* with respect to the world reference frame Σ_*O*_, *S*(*c*_*j*_ − *p*_*ts*_) is the cross-product matrix of the vector $\bar{c_{j}p_{ts}}$ , and *p*_*ts*_ is center of mass of the grasped portion of tissue. Accounting the available grasping configurations, we can refine the general formula as follows:

$$\lambda =\left\{\begin{array}{cc} N_{G}\xi & \text{if method}=Po\\ -G^{†}\omega +N_{G}\xi & \text{if method}=Pr\\ -G^{†}\omega & \text{if method}=Rt \end{array}\right.$$ (40)

At each time instant, the position of the centroid of the manipulated tissues and the positions of the contact points may be known, so the grasping matrix can be computed and a projector in its null space can be evaluated:

$$N_{G}=I-G^{†}G.$$ (41)

Thus, given measure of contact force, $\tilde{\lambda }$ , the value of *ξ* and *ω* can be evaluated as:

$$\xi =\left\{\begin{array}{cc} N_{G}^{-1}\tilde{\lambda } & \text{if method}=Po\\ N_{G}^{†}\tilde{\lambda } & \text{if method}=Pr\\ 0 & \text{if method}=Rt \end{array}\right.$$ (42)

$$\omega =\left\{\begin{array}{cc} -{\left(G^{†}\right)}^{†}\tilde{\lambda } & \text{if method}\neq Po\\ 0 & \text{if method}=Po \end{array}\right.$$ (43)

### 3.2 Lyapunov Stability Proof

The stability of the proposed control law was assessed by Lyapunov conditions. To this aim, we choose as positive definite Lyapunov functions the function of error:

$$V=\frac{1}{2}\epsilon^{T}K_{c}^{T}\epsilon .$$ (44)

Where ϵ is the distance between the actual (*p*_*r*_) and desired ( $\tilde{p_{r}}$ ) MUSHA fingertip.

$$\epsilon ={\tilde{p}}_{r}-p_{r}$$ (45)

It is possible to evaluate its time derivative value as:

$$\dot{\epsilon }=\dot{\tilde{p_{r}}}-\hat{J_{r}^{w}}\dot{q_{r}}$$ (46)

Consequently, the time derivative of the Lyapunov function is

$$\dot{V}=\epsilon^{T}K_{c}^{T}\left(\dot{\tilde{p_{r}}}-\hat{J_{r}^{w}}\dot{q_{r}}\right).$$ (47)

Where $\hat{J_{r}^{w}}$ is the Jacobian matrix of model of MUSHA hand evaluated in real current position. Substituting (28) in (47), the derivative Lyapunov function becomes

$$\dot{V}=\epsilon^{T}K_{c}^{T}\dot{\tilde{p_{r}}}-\epsilon^{T}K_{c}^{T}\hat{J_{r}^{w}}\left[J_{r}^{w†}\left[M\dot{p_{h}}+J_{r}^{w}N_{M}u+K_{c}\left({\tilde{p}}_{r}-p_{r}\right)\right]\right].$$ (48)

Where M is the mapping action of control law

$$M=J_{rs}\left(p_{r}\right)K_{s}J_{hs}^{†}\left(p_{h}\right)I\Omega ,$$ (49)

and $N_{M}$ is the projection action in null space of M used to overcome the duality of thumb pose in retractor method:

$$N_{M}=I-{\left(J_{rs}\left(p_{r}\right)K_{s}J_{hs}^{†}\left(p_{h}\right)I\Omega \right)}^{†}\left(J_{rs}\left(p_{r}\right)K_{s}J_{hs}^{†}\left(p_{h}\right)I\Omega \right).$$ (50)

The desired velocity ${\dot{\tilde{p}}}_{r}$ is chosen as the result of mapping $M\left(q_{r},p_{r},p_{h}\right)$ and projection in null space of mapping $N_{M}$ ,

$${\dot{\tilde{p}}}_{r}=M{\dot{p}}_{h}+J_{r}^{w}N_{M}u.$$ (51)

Introducing (48) in (51), it is obtained that:

$$\dot{V}=\epsilon^{T}K_{c}^{T}\dot{\tilde{p_{r}}}-\epsilon^{T}K_{c}^{T}\hat{J_{r}^{w}}J_{r}^{w†}\left[\dot{\tilde{p_{r}}}+K_{c}\left({\tilde{p}}_{r}-p_{r}\right)\right].$$ (52)

Reintroducing (52) the error evaluated in accordance with (45), we obtain

$$\dot{V}=\epsilon^{T}K_{c}^{T}\dot{\tilde{p_{r}}}-\epsilon^{T}K_{c}^{T}\hat{J_{r}^{w}}J_{r}^{w†}\left(\dot{\tilde{p_{r}}}+K_{c}\epsilon \right).$$ (53)

If MUSHA is accurately calibrated the Jacobian matrix evaluated on the real position of fingers and on the estimated one are approximately equal, $\hat{J_{r}^{w}}\approx J_{r}^{w}$ , and the time derivative Lyapunov function becomes

$$\dot{V}\approx -\epsilon^{T}K_{c}^{T}K_{c}\epsilon .$$ (54)

Since the Lyapunov function (47) is positive definite (*V*(*t*) > 0) and its time derivative is negative definite $\left(\dot{V}\left(t\right)<0\right)$ , the system is Lyapunov stable.

It is possible assert the BIBO stability, too. In fact, substituting definition of desired position (51) in control law (28), we obtain that

$${\dot{q}}_{r}=J_{r}^{w†}\left({\dot{\tilde{p}}}_{r}-K_{c}\epsilon \right)$$ (55)

It is possible to evaluate the tendency of error, using the definition of time derivative of error metric:

$$\dot{\epsilon }=K_{c}\epsilon$$ (56)

The error evolves as first order linear system, so the Lyapunov stability implies the BIBO stability.

### 3.3 User Studies

**FIGURE 8:**
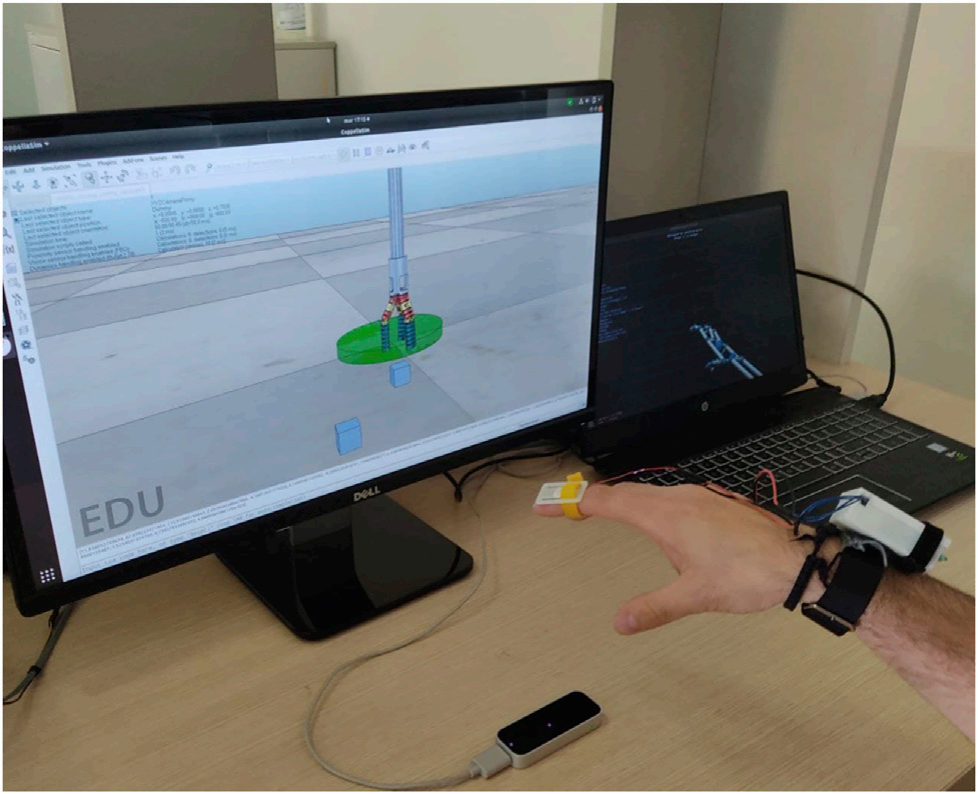
The experimental set-up consisted in the LeapMotion controller, the haptic bracelet and a couple of screen to visualize the Virtual environment and the reconstructed model of tracked hand. In the virtual scene, cubes pseudo-randomly changed their position at the beginning of the single repetition of experiment.

Ten inexperienced participants (six males and four females, aged 24–59, all right-handed) took part in user studies. Each subject gave her/his consent to participate and was able to discontinue participation at any time during experiments. The experimental evaluation protocols followed the declaration of Helsinki, and there was no risk of harmful effects on subjects’ health. Participants performed 10 pick-and-place tasks of two cubes with 10 different initial scenarios pseudo-randomly sorted. The experimental set-up is shown in^1^ Figure 8. The dimension of each cube is 0.02 m × 0.02 m×0.02 m and the weight is equal to 20 g. To help the participant to complete the task, force feedback is provided by two vibromotors NFP-C1234L to inform them about internal and external forces applied on cubes. The two components of force are evaluated starting from the results of Section 3.1 using (42) and (43), respectively, and their norms in *ℓ* ^2^ space, ‖*ξ*‖ and ‖*ω*‖, are used to calculate the motor PWMs as:

$$PWM_{\xi }=\left\lceil \frac{1}{k}\frac{\left\|\xi \right\|}{\Delta F}255\right\rceil ,$$ (57)

$$PWM_{\omega }=\left\lceil \frac{\left\|\omega \right\|}{\Delta F}255\right\rceil .$$ (58)

where Δ*F* is the range of forces that can be acquired through MUSHA hand force sensors (Δ*F* = 4*N*). The dimension disparity between the internal force space ( $\xi \in R^{18}$ ) and the external one ( $\omega \in R^{6}$ ) introduces an imbalance of haptic stimulus intensity which has been compensated by scaling the *ℓ* ^2^ norm ‖*ξ*‖ with a *k*–factor equal to the ratio of the dimensions of the two spaces (*k* = 3).

At the end of each session, a NASA Task Load Index questionnaire (TLX) (Hart, 2006) was proposed to the participants, with the aim of assessing the perceived load in terms of Mental Demand (MD), Temporal Demand (TD), Physical Demand (PD), Performance (PE), Effort (EF), and Frustration (FR). This method assesses workload on a point-based scale. Each of the six questions has a scale of 21 levels, considering 1 as “very low” and 21 as “very high.” Questions are reported in what follows:

- Mental demand: *“How mentally demanding was the task?”*
- Physical demand: *“How physically demanding was the task?”*
- Temporal demand: *“How hurried or rushed was the pace of the task?”*
- Performance: *“How successful were you in accomplishing what you were asked to do?”*
- Effort: *“How hard did you have to work to accomplish your level of performance?”*
- Frustration: *“How insecure, discouraged, irritated, stressed, and annoyed were you?”*

All participants successfully completed the ten pick and place repetitions of the two cubes, so the completion times for each trial and the results of the questionnaires were considered as metrics for evaluation. Results are reported in Table 1 and Table 2. The mean time to complete the task is 16.49 s and no participant took more than 30s. The load of the task performed with the proposed control law is evaluated from answers of the NASA TLX questionnaire and its mean value is equal to 35%. According with the proposed task and experimental set-up we used the following weights: *w*_*MD*_ = 3, *w*_*PD*_ = 0, *w*_*TD*_ = 5, *w*_*PE*_ = 1, *w*_*FR*_ = 3, *w*_*EF*_ = 3. The physical demand was excluded from task load evaluation (PD = 0) because the physical load is due to the absence of arm support that is provided by the surgeon console in a real scenario.

**TABLE 1:** Time to complete tasks (mean and ±std).

User #	1	2	3	4	5	6	7	8	9	10
Means (s)	21.5	23.9	25.3	12.6	11.80	14.0	16.9	12.2	12.2	14.0
Std (s)	±4.3	±6.9	±6.3	±2.3	±2.0	±4.7	±3.9	±2.2	±2.5	±4.7

**TABLE 2:** NASA TLX questionnaire results, tally of importance selections: *w*_*MD*_ = 3, *w*_*PD*_ = 0, *w*_*TD*_ = 5, *w*_*PE*_ = 1, *w*_*FR*_ = 3, *w*_*EF*_ = 3.

User #	1	2	3	4	5	6	7	8	9	10
MD	6	8	5	10	8	6	8	5	5	6
PD	10	7	12	18	13	6	5	12	6	6
TD	3	4	2	7	8	11	13	9	5	7
PE	8	4	2	5	6	6	6	5	5	6
EF	12	12	10	13	11	6	7	5	6	5
FR	12	12	10	9	7	2	10	5	2	2
Load	7.5	8.2	6.4	9.1	8.2	6.8	12.2	6.3	4.9	5.5

## 4 Discussion

In this work, we present a reliable and intuitive method for the bilateral asymmetric teleoperation of the MUSHA hand. A camera-based device is in charge of tracking the human hand, and a mapping algorithm projects the human motion in the end-effector actuation space through the virtual object domain. The developed technique consists in a remote control for the projection of the human gestures into the robot space of actuation by crossing the object domain and switching in the function of the current gripping method, which is recognized online by the posture of the user’s hand. A sphere is chosen as virtual objects due to the reduced number of parameters that describe its pose and deformation. A closed-loop inverse kinematic algorithm makes more robust the control action and guarantees the BIBO stability of the algorithm, while a projection in the null of the object space allows the correct estimation of indeterminate poses. Starting from the kinematics description of the MUSHA hand and its main grasping methods, we theoretically evaluated the grasping forces applied by the tool during each method and an experimental campaign assesses the functionality and the usability of the algorithm. All the participants completed the assigned task in less than 30 s (15 s per cube) with a limited cognitive load. The obtained results demonstrate the intuitiveness of the teleoperation system.

The proposed control algorithm has been designed, developed, and tested to control the MUSHA hand, nevertheless it can be generalized and extended to different grippers and robotic hands. Indeed the proposed methodology relies on a mapping algorithm that exploits contact points and grasping methods. Thus, a redefinition of the differential kinematic equations and of the related Jacobian Matrix of the gripper allow the reader adapting the algorithm to the hand of interest. A preliminary analysis of the instrument grasping capacity and the redefinition of the selection vector, Ω, allows to maintain the validity of the theoretical results regarding the evaluation of the applied forces. In future work, experiments will be conducted considering expert users asking them to complete elementary surgical procedures using the real MUSHA hand mounted on the da Vinci robot and we will test the compatibility of the algorithm with several standard end-effector during the same procedures. Finally, the entire system will be integrated with the surgeon console of the da Vinci robot.

## Data Availability

The raw data supporting the conclusion of this article will be made available by the authors, without undue reservation.

## Footnotes

1 A video demonstration of the experimental task is available at the following link: https://youtu.be/6DYI1MfqI7A.
