# Supplementary material for: A Human Gesture Mapping Method to Control a Multi‐Functional Hand for Robot‐Assisted Laparoscopic Surgery: The MUSHA Case
Source: Front Robot AI. 2021 Dec 10;8:741807. doi: 10.3389/frobt.2021.741807 (PMC8725602; doi:10.3389/frobt.2021.741807)
Supplement: Supplementary file 1 [file Table1.pdf]

1 SUPPLEMENTARY MATERIAL

1.1 MUSHA Full Kinematic Model

The full kinematic model of MUSHA hand is equal to:

$$
\begin{aligned}
x_{r,T} &= \cos(q_W)(a_{2,T}\cos(q_{2,T}) + a_{3,T}\cos(q_{3,T})) \\
y_{r,T} &= \sin(q_W)(a_{2,T}\cos(q_{2,T}) + a_{3,T}\cos(q_{3,T})) \\
z_{r,T} &= a_{2,T}\sin(q_{2,T}) + a_{3,T}\sin(q_{3,T}) \\
a_{2,T} &= \begin{cases} (\frac{6L_{2,T}}{5q_{2,T}})sin(\frac{5q_{2,T}}{6}) \\ L_{2,T} \text{ if } q_{2,T} = 0 \end{cases} \\
a_{3,T} &= \begin{cases} (\frac{6L_{3,T}}{5q_{3,T}})sin(\frac{5q_{3,T}}{6}) \\ L_{3,T} \text{ if } q_{3,T} = 0 \end{cases} \\
x_{r,I} &= \cos(q_W + \frac{2\pi}{3})(a_{PIP}\cos(q_{PIP}) + a_{3,I}\cos(q_{3,I})) \\
y_{r,I} &= \sin(q_W + \frac{2\pi}{3})(a_{PIP}\cos(q_{PIP}) + a_{3,I}\cos(q_{3,I})) \\
z_{r,I} &= a_{PIP}\sin(q_{PIP}) + a_{3,I}\sin(q_{3,I}) \\
a_{PIP} &= \begin{cases} (\frac{6L_{PIP}}{5q_{PIP}})\sin(\frac{5q_{PIP}}{6}) \\ L_{PIP} \text{ if } q_{PIP} = 0 \end{cases} \\
a_{3,I} &= \begin{cases} (\frac{6L_{3,I}}{5q_{3,I}})sin(\frac{5q_{3,I}}{6}) \\ L_{3,I} \text{ if } q_{3,I} = 0 \end{cases} \\
x_{r,M} &= \cos(q_W + \frac{4\pi}{3})(a_{PIP}\cos(q_{PIP}) + a_{3,M}\cos(q_{3,M})) \\
y_{r,M} &= \sin(q_W + \frac{4\pi}{3})(a_{PIP}\cos(q_{PIP}) + a_{3,M}\cos(q_{3,M})) \\
z_{r,M} &= a_{PIP}\sin(q_{PIP}) + a_{3,M}\sin(q_{3,M}) \\
a_{3,M} &= \begin{cases} (\frac{6L_{3,M}}{5q_{3,M}})\sin(\frac{5q_{3,M}}{6}) \\ L_{3,M} \text{ if } q_{3,M} = 0 \end{cases}
\end{aligned} \tag{1}
$$

1.2 MUSHA Differential Model

The full differential kinematic model of MUSHA hand is equal to $\dot{p}_r = J_r \dot{q}_r$ with:

$$
\begin{aligned}
J_{r11} &= -s_W \frac{6}{5}\left(\frac{1}{q_{2,T}} s_{\frac{5}{6}2,T} c_{2,T} + \frac{1}{q_{3,T}} s_{\frac{5}{6}3,T} c_{3,T}\right) \\
J_{r12} &= c_W \frac{6}{5}\left(\frac{1}{q_{2,T}}\left(c_{2,T}\frac{5}{6} c_{\frac{5}{6}2,T} - s_{2,T} s_{\frac{5}{6}2,T}\right) - \frac{1}{q_{2,T}^2} c_{2,T} s_{\frac{5}{6}2,T}\right) \\
J_{r13} &= c_W \frac{6}{5}\left(\frac{1}{q_{3,T}}\left(c_{3,T}\frac{5}{6} c_{\frac{5}{6}3,T} - s_{3,T} s_{\frac{5}{6}3,T}\right) - \frac{1}{q_{3,T}^2} c_{3,T} s_{\frac{5}{6}3,T}\right) \\
J_{r21} &= c_W \frac{6}{5}\left(\frac{1}{q_{2,T}} s_{\frac{5}{6}2,T} c_{2,T} + \frac{1}{q_{3,T}} s_{\frac{5}{6}3,T} c_{3,T}\right) \\
J_{r22} &= s_W \frac{6}{5}\left(\frac{1}{q_{2,T}}\left(s_{2,T} s_{\frac{5}{6}2,T} - c_{2,T}\frac{5}{6} c_{\frac{5}{6}2,T}\right) + \frac{1}{q_{2,T}^2} c_{2,T} s_{\frac{5}{6}2,T}\right) \\
J_{r23} &= s_W \frac{6}{5}\left(\frac{1}{q_{3,T}}\left(s_{3,T} s_{\frac{5}{6}3,T} - c_{3,T}\frac{5}{6} c_{\frac{5}{6}3,T}\right) + \frac{1}{q_{3,T}^2} c_{3,T} s_{\frac{5}{6}3,T}\right) \\
J_{r32} &= c_{2,T}\frac{5}{6q_{2,T}} s_{\frac{5}{6}2,T} + s_{2,T}\left(\frac{1}{q_{2,T}} c_{\frac{5}{6}2,T} - \frac{6}{5q_{2,T}^2} s_{\frac{5}{6}2,T}\right) \\
J_{r33} &= c_{3,T}\frac{5}{6q_{3,T}} s_{\frac{5}{6}3,T} + s_{3,T}\left(\frac{1}{q_{3,T}} c_{\frac{5}{6}3,T} - \frac{6}{5q_{3,T}^2} s_{\frac{5}{6}3,T}\right) \\
J_{r41} &= -s_{W+\frac{2\pi}{3}} \frac{6}{5}\left(\frac{1}{q_{PIP}} s_{\frac{5}{6}2,I} c_{PIP} + \frac{1}{q_{3,I}} s_{\frac{5}{6}3,I} c_{3,I}\right) \\
J_{r44} &= c_{W+\frac{2\pi}{3}} \frac{6}{5}\left(\frac{1}{q_{PIP}}\left(c_{PIP}\frac{5}{6} c_{\frac{5}{6}PIP} - s_{PIP} s_{\frac{5}{6}PIP}\right)\right) - c_{W+\frac{2\pi}{3}} \frac{6}{5}\left(\frac{1}{q_{PIP}^2} c_{PIP} s_{\frac{5}{6}PIP}\right) \\
J_{r45} &= c_{W+\frac{2\pi}{3}} \frac{6}{5}\left(\frac{1}{q_{3,I}}\left(c_{3,I}\frac{5}{6} c_{\frac{5}{6}3,I} - s_{3,I} s_{\frac{5}{6}3,I}\right) - \frac{1}{q_{3,I}^2} c_{3,I} s_{\frac{5}{6}3,I}\right) \\
J_{r51} &= c_{W+\frac{2\pi}{3}} \frac{6}{5}\left(\frac{1}{q_{PIP}} s_{\frac{5}{6}p} c_{PIP} + \frac{1}{q_{3,I}} s_{\frac{5}{6}3,I} c_{3,I}\right) \\
J_{r54} &= s_{W+\frac{2\pi}{3}} \frac{6}{5}\left(\frac{1}{q_{PIP}}\left(s_{PIP} s_{\frac{5}{6}PIP} - c_{PIP}\frac{5}{6} c_{\frac{5}{6}PIP}\right)\right) + s_{W+\frac{2\pi}{3}} \frac{6}{5}\left(\frac{1}{q_{PIP}^2} c_{PIP} s_{\frac{5}{6}PIP}\right) \\
J_{r55} &= s_{W+\frac{2\pi}{3}} \frac{6}{5}\left(\frac{1}{q_{3,I}}\left(s_{3,I} s_{\frac{5}{6}3,I} - c_{3,I}\frac{5}{6} c_{\frac{5}{6}3,I}\right) + \frac{1}{q_{3,I}^2} c_{3,I} s_{\frac{5}{6}3,I}\right) \\
J_{r65} &= c_{3,I}\frac{5}{6q_{3,I}} s_{\frac{5}{6}3,I} + s_{3,I}\left(\frac{1}{q_{3,I}} c_{\frac{5}{6}3,I} - \frac{6}{5q_{3,I}^2} s_{\frac{5}{6}3,I}\right) \\
J_{r71} &= -s_{W+\frac{4\pi}{3}} \frac{6}{5}\left(\frac{1}{q_{2,M}} s_{\frac{5}{6}2,M} c_{2,M} + \frac{1}{q_{3,M}} s_{\frac{5}{6}3,M} c_{3,M}\right) \\
J_{r74} &= c_{W+\frac{4\pi}{3}} \frac{6}{5}\left(\frac{1}{q_{PIP}}\left(c_{PIP}\frac{5}{6} c_{\frac{5}{6}PIP} - s_{PIP} s_{\frac{5}{6}PIP}\right)\right) - c_{W+\frac{4\pi}{3}} \frac{6}{5}\left(\frac{1}{q_{PIP}^2} c_{PIP} s_{\frac{5}{6}PIP}\right) \\
J_{r76} &= c_{W+\frac{4\pi}{3}} \frac{6}{5}\frac{1}{q_{3,M}}\left(c_{3,M}\frac{5}{6} c_{\frac{5}{6}3,M} - s_{3,M} s_{\frac{5}{6}3,M}\right) - c_{W+\frac{4\pi}{3}} \frac{6}{5}\frac{1}{q_{3,M}^2} c_{3,M} s_{\frac{5}{6}3,M} \\
J_{r81} &= c_{W+\frac{4\pi}{3}} \frac{6}{5}\frac{1}{q_{PIP}} s_{\frac{5}{6}p} c_{PIP} + \frac{1}{q_{3,M}} s_{\frac{5}{6}3,M} c_{3,M} \\
J_{r84} &= s_{W+\frac{4\pi}{3}} \frac{6}{5}\frac{1}{q_{PIP}}\left(s_{PIP} s_{\frac{5}{6}PIP} - c_{PIP}\frac{5}{6} c_{\frac{5}{6}PIP}\right) + c_{W+\frac{4\pi}{3}} \frac{6}{5}\frac{1}{q_{PIP}^2} c_{PIP} s_{\frac{5}{6}PIP} \\
J_{r86} &= s_{W+\frac{4\pi}{3}} \frac{6}{5}\frac{1}{q_{3,M}}\left(s_{3,M} s_{\frac{5}{6}3,M} - c_{3,M}\frac{5}{6} c_{\frac{5}{6}3,M}\right) + s_{W+\frac{4\pi}{3}} \frac{6}{5}\frac{1}{q_{3,M}^2} c_{3,M} s_{\frac{5}{6}3,M} \\
J_{r94} &= J_{64} = c_{PIP}\frac{5}{6q_{PIP}} s_{\frac{5}{6}PIP} + s_{PIP}\left(\frac{1}{q_{PIP}} c_{\frac{5}{6}PIP} - \frac{6}{5q_{PIP}^2} s_{\frac{5}{6}2p}\right) \\
J_{r96} &= c_{3,M}\frac{5}{6q_{3,M}} s_{\frac{5}{6}3,M} + s_{3,M}\left(\frac{1}{q_{3,M}} c_{\frac{5}{6}3,M} - \frac{6}{5q_{3,M}^2} s_{\frac{5}{6}3,M}\right)
\end{aligned} \tag{2}
$$

other terms of jacobian matrix are null.
